# YAP activation in Müller cells protects against NMDA-induced retinal ganglion cell injury by regulating Bcl-xL expression

**DOI:** 10.3389/fphar.2024.1446521

**Published:** 2024-08-06

**Authors:** Toshihide Kashihara, Yui Morita, Misaki Hatta, Sae Inoue, Yume Suzuki, Akane Morita, Tsutomu Nakahara

**Affiliations:** Department of Molecular Pharmacology, Kitasato University School of Pharmaceutical Sciences, Tokyo, Japan

**Keywords:** retina, Müller cell, retinal ganglion cell, YAP, Bcl-XL, NMDA, mitochondrial dysfunction

## Abstract

Retinal neurodegeneration, characterized by retinal ganglion cell (RGC) death, is a leading cause of vision impairment and loss in blind diseases, such as glaucoma. Müller cells play crucial roles in maintaining retinal homeostasis. Thus, dysfunction of Müller cells has been implicated as one of the causes of retinal diseases. Yes-associated protein 1 (YAP), a nuclear effector of the Hippo pathway, regulates mammalian cell survival. In this study, we investigated the role of YAP in Müller cells during *N*-methyl-D-aspartic acid (NMDA)-induced excitotoxic RGC injury in rats. We found that YAP expression increased and was activated in Müller cells after NMDA-induced RGC injury. This YAP response was partly due to an increase in *Yap* mRNA levels, although it may be independent of the Hippo pathway and β-TrCP-mediated YAP degradation. Morphological analysis revealed that verteporfin, a selective YAP inhibitor, exacerbated NMDA-induced RGC degeneration, suggesting that YAP activation in Müller cells contributes to RGC survival in NMDA-treated retinas. Studies in the rat Müller cell line (rMC-1) demonstrated that overexpression of YAP increased the levels of Bcl-xL, while verteporfin decreased the levels of Bcl-xL and cell viability and increased the levels of cytochrome c released from mitochondria and cleaved caspase-3. Finally, we found that Bcl-xL expression increased slightly in NMDA-treated retinas, whereas intravitreal injection of verteporfin suppressed this increase. Our findings suggest that activated YAP in Müller cells protects against NMDA-induced RGC injury by upregulating Bcl-xL expression.

## 1 Introduction

Retinal neurodegeneration, characterized by retinal ganglion cell (RGC) death, is a leading cause of vision impairment and loss in blind diseases such as glaucoma (Gupta and Yücel, 2007; Schmidt et al., 2008; Kaur and Singh, 2021). Glutamate is the primary excitatory neurotransmitter in the central nervous system, including the retina. Excitotoxicity, which is triggered by the excessive stimulation of *N*-methyl-D-aspartic acid (NMDA) receptors by glutamate, is one of the mechanisms of neurodegeneration in the retina and brain (Gupta and Yücel, 2007; Lewerenz and Maher, 2015). Therefore, NMDA-induced excitotoxicity, an acute RGC injury model, has been used to study the mechanisms underlying retinal neurodegeneration and neuroprotection (Lam et al., 1999; Lambuk et al., 2019).

Healthy Müller cells span the entire thickness of the retina and interact with all retinal neurons and blood vessels (Bringmann et al., 2006). They play essential roles in regulating the function and metabolism of neurons, the retinal extracellular milieu, redox balance, and retinal blood flow (Bringmann et al., 2006; Reichenbach and Bringmann, 2013). However, Müller cells undergo reactivation (gliosis) in response to virtually all pathological retinal stimulations (Bringmann et al., 2006; Bringmann and Wiedemann, 2012). Reactive Müller cells protect retinal neurons but may also stop supporting the neurons and accelerate the progress of retinal neurodegeneration (Bringmann et al., 2006; Bringmann and Wiedemann, 2012). Thus, a better understanding of the Müller cell responses in retinal neurodegeneration could facilitate the development of novel therapeutic strategies for retinal diseases.

Yes-associated protein 1 (YAP), a transcriptional coactivator and downstream effector of the Hippo pathway, is a key regulator of cell survival, proliferation, and metabolism in multiple organs, including the eye (Yu et al., 2015; Koo and Guan, 2018; Lee et al., 2018). YAP activity is primarily regulated through inhibitory phosphorylation by Hippo pathway kinases, large tumor suppressor kinases 1 and 2 (LATS1/2). When the Hippo pathway is turned off, YAP accumulates in the nucleus, stimulating the expression of target genes such as Cyr61, Bcl-2, and Bcl-xL (LeBlanc et al., 2018; Nguyen and Yi, 2019). Previous studies have shown that Hippo-YAP signaling plays a critical role in eye development (Lee et al., 2018). In the adult retina, YAP is normally expressed in Müller cells, whereas its expression is low in retinal neurons (Hamon et al., 2017; Lee et al., 2018; Masson et al., 2020). The effect of YAP loss-of-function in Müller cells demonstrates that YAP downregulation causes Müller cell dysfunction and results in retinal neurodegeneration, indicating that YAP in Müller cells is essential for the maintenance of retinal homeostasis and the preservation of retinal neurons (Masson et al., 2020; Yang et al., 2022). Furthermore, it has been reported that YAP signaling is upregulated in response to photoreceptor degeneration (Hamon et al., 2017). However, the exact function of YAP in Müller cells remains unclear.

To elucidate the role of YAP in Müller cells in RGC degeneration, we investigated the changes in expression levels of YAP and its function in Müller cells after NMDA-induced retinal injury in rats.

## 2 Materials and methods

### 2.1 Animals

Male Sprague-Dawley rats (7–8 weeks old; Oriental Yeast Co., Tokyo, Japan) were used in the experiments. The rats were housed in a temperature-controlled environment within a 20°C–24°C range with a 12-h light/dark cycle and were given free access to water and standard diet. All experiments involving animals were approved by the Institutional Animal Care and Use Committee of Kitasato University (approval no. 20-45 and 23-7). All animal procedures and experiments were performed in accordance with the Association for Research in Vision and Ophthalmology Statement for the Use of Animals in Ophthalmic and Vision Research (ARVO).

### 2.2 Intravitreal injection

Intravitreal injections were performed with a 32-gauge needle mounted on a 25-μL Hamilton syringe (Hamilton Company), as previously described (Watanabe et al., 2021). Briefly, rats were anesthetized by subcutaneous injection of 0.25 mg/kg medetomidine (Medetomin, Meiji Seika Pharma, Japan), 1.35 mg/kg midazolam (Teva Takeda Pharma, Japan), and 1.65 mg/kg butorphanol (Vetorphale, Meiji Seika Pharma, Japan).

To determine whether YAP is activated in the retina when NMDA induces the most significant damage to RGCs, NMDA (200 nmol; Nacalai Tesque), which was dissolved in dimethyl sulfoxide (DMSO), at a total volume of 5 μL was injected into the vitreous cavity of one eye (Manabe and Lipton, 2003; Asami et al., 2015). An equal volume of DMSO was injected into the vitreous cavity of the other eye as a control.

To determine the effects of verteporfin (VP), a selective YAP inhibitor (Liu-Chittenden et al., 2012), on retinal structure, the rats were divided into four groups: DMSO + DMSO, DMSO + VP, NMDA + DMSO, and NMDA + VP. First, DMSO or NMDA (20 or 200 nmol/eye) at a total volume of 5 μL was injected into the vitreous cavity of both eyes in each rat. Two days after the injection, VP (3 nmol/eye) at a total volume of 5 μL was injected into the vitreous cavity of one eye. As a control for VP, an equal volume of DMSO was injected into the vitreous cavity of the other eye. We used this dose of VP from our data, showing that intravitreal injection of this dose of VP alone had minimal impact on the retina.

At the indicated time points after injection, the rats were deeply anesthetized by subcutaneous injection of 0.72 mg/kg medetomidine, 6.66 mg/kg midazolam, and 4.65 mg/kg butorphanol, and their eyes were enucleated and processed for histological analysis, immunohistochemistry, Western blotting, and RNA extraction, according to the procedures described below.

### 2.3 Immunohistochemistry and terminal deoxynucleotidyl transferase-mediated deoxyuridine triphosphate nick-end labeling (TUNEL)

Immunohistochemical staining of frozen rat retinal cross-sections was performed as previously described with some modifications (Watanabe et al., 2021). Briefly, rats were deeply anesthetized and systemic perfusion was performed with 1% paraformaldehyde (PFA; Nacalai Tesque) in phosphate-buffered saline (PBS) through the aorta. Their eyes were enucleated and the eyecups without the lens were further fixed in 1%PFA/PBS solution for 60 min at 4°C. The eyecups were incubated in 15% and 30% sucrose/PBS solutions and frozen in an optimum cutting-temperature (OCT) compound (Sakura Finetek, Japan). The eyecups were sectioned through the optic disc using a cryostat at 16 μm thickness. The sections were washed 3 times with PBS containing 0.3% Triton X-100 (PBST), incubated in blocking solution (Blocking One Histo, Nacalai Tesque) for 10 min, and then incubated with the following primary antibodies in Signal Booster Immunostain solution M (Beacle Inc.) overnight at 4°C: rabbit monoclonal anti-the active form of YAP (active-YAP) antibody (1:500, ab205270, abcam), mouse monoclonal anti-glutamine synthetase (GS) antibody (1:800, MAB302, Sigma-Aldrich), rabbit monoclonal anti-Bcl-xL antibody [1:200, 2764, Cell Signaling Technology (CST)], and mouse monoclonal anti-4-hydroxy-2-nonenal (4-HNE) antibody (1:100, MHN-100P, Japan Institute for the Control of Aging). After washing five times with PBST, the sections were incubated with Alexa Fluor 488-conjugated AffiniPure donkey anti-rabbit IgG (H + L) (1:400, 711–545–152; Jackson ImmunoResearch Laboratories) or Cy3-conjugated AffiniPure donkey anti-mouse IgG (H + L) (1:400, 715–165–151; Jackson ImmunoResearch Laboratories) in Signal Booster Immunostain solution M for 60 min at room temperature. The sections were then washed in PBST and mounted using the Vectashield Mounting Medium with DAPI (H-1200, Vector Laboratories). Fluorescence images were captured using a fluorescence microscope (BZ-9000; Keyence).

TUNEL staining was performed to detect apoptotic cells using a TUNEL kit (*In Situ* Cell Death Detection kit, Fluorescein, 11684795910, Roche) according to the manufacturer’s instructions. Briefly, the frozen retinal sections were incubated with the TUNEL kit for 60 min at 37°C, and the nuclei were stained using the Vectashield Mounting Medium with DAPI. Fluorescence images were captured using a fluorescence microscope (BZ-9000); five images were obtained for each sample. The numbers of TUNEL-positive cells in the ganglion cell layer (GCL) and the inner nuclear layer (INL) were measured for each image. The average number of TUNEL-positive cells in each layer was then calculated.

### 2.4 Histological evaluation of the retina

Histological evaluation was performed as described previously (Watanabe et al., 2021). Briefly, 7 days after intravitreal injection, the rats were deeply anesthetized. The eyes were enucleated, fixed in Davidson’s solution, dehydrated, and embedded in paraffin. The eyecups were sectioned through the optic disc using a microtome at 5 μm thickness. The sections were stained with hematoxylin and eosin (HE). The cell counting in the GCL and the measurement of the inner plexiform layer (IPL), the INL, the outer plexiform layer (OPL), and the outer nuclear layer (ONL) thicknesses were performed in a region 1,250–1,500 μm from the center of the optic nerve head on both sides. The average number of cells per eye was then calculated.

### 2.5 Adenovirus construction

FLAG-YAP adenovirus (Ad-YAP) and LacZ control adenovirus (Ad-LacZ) were generated as described previously (Del et al., 2013; Kashihara et al., 2022). Briefly, the pBHGloxΔE1,3Cre plasmid was co-transfected with the pDC316 shuttle vector (Microbix Biosystems) containing the gene of interest into HEK293 cells (American Type Culture Collection) using Lipofectamine 2000 (Thermo Fisher Sciences).

### 2.6 Cell culture and preparations

Rat retinal Müller cells (rMC-1; Kerafast Inc., MA, United States) were maintained at 37°C with 5% CO_2_ in DMEM (08459–35, Nacalai Tesque) supplemented with 10% fetal bovine serum (FBS) and penicillin/streptomycin (3553–74, Nacalai Tesque). The rMC-1 cells were passaged once every 2 or 3 days. For Western blotting, rMC-1 cells were seeded at approximately 100% confluence in 6-well plastic dishes to minimize the impact of cell proliferation and were cultured overnight. After changing to a new medium, the cells were transduced with Ad-LacZ or Ad-YAP in DMEM supplemented with 1%FBS (Kashihara et al., 2022) or treated with DMSO or VP (0.3–3 μM) in DMEM supplemented with 10%FBS for 3 days. For the MTT assay, rMC-1 cells were seeded at approximately 100% confluence in 96-well plates to minimize the impact of cell proliferation and were cultured overnight. After changing to the medium containing with DMSO or VP (0.3–3 μM), cells were cultured for 3 days.

### 2.7 Western blotting

Retinas, which were isolated from rat eyes, and rMC-1 cells were lysed with ice-cold lysis buffer (10 mM Tris pH 7.5, 150 mM NaCl, 5 mM EDTA, 1% Triton X-100, 50 mM NaF, and 10% glycerol) containing protease inhibitor (M8699, Sigma-Aldrich) and 1 μM MG-132 (Sigma-Aldrich) (Kashihara et al., 2022). The release of cytochrome c from mitochondria was measured using the cytoplasmic fraction homogenized gently with a BioMasher II (Funakoshi) and centrifuged at 10,000 g for 20 min. Protein concentrations were measured using a BCA protein assay kit (Nacalai Tesque, Kyoto, Japan). Denatured protein samples (10–20 μg) were separated by 10% or 12% SDS-PAGE and transferred to a polyvinylidene difluoride membrane using a Trans-Blot Turbo system (Bio-Rad). Non-specific binding was blocked using Bloking One or Bloking One-P (Nacalai Tesque, Kyoto, Japan) for 30 min at room temperature. After being rinsed with Tris-buffered saline containing 0.5% Tween 20 (TBST), the membranes were incubated with primary antibodies against total-YAP (1:2000, 14074, CST), active-YAP (1:2000, ab205270, abcam), pS127-YAP (1:2000, 4911, CST), LATS1 (1:2000, 3477, CST), pS909-LATS1 (1:2000, 9157, CST), β-transducin repeat-containing protein (β-TrCP) (1:2000, 4394, CST), glyceraldehyde3-phosphate dehydrogenase (GAPDH) (1:5000, 2118, CST), Cyr61 (1:2000, 39382, CST), Bcl-xL (1:2000, 2764, CST), Bax (1:2000, 14796, CST), cleaved caspase-3 (1:1,500, 9664, CST), and cytochrome c (1:2000, 11940, CST). After washing with TBST, the membranes were incubated with horseradish peroxidase (HRP)-conjugated secondary antibodies (1:5000, 7076, CST) for 60 min at room temperature. The bound secondary antibodies were visualized using Chemi-Lumi One (Nacalai Tesque), Immobilon Western Chemiluminescent HRP Substrate (Merck), or Immobilon ECL Ultra Western Chemiluminescent HRP Substrate (Merck), using a ChemiDoc Touch MP system (Bio-Rad). Band signal intensities were quantified using ImageJ (NIH, United States). Data were normalized to GAPDH expression levels.

### 2.8 RNA extraction and RT-qPCR

Total RNA was extracted from a single retina using the TRIzol reagent (Thermo Fisher Scientific). cDNA was generated using 600 ng total RNA and PrimeScript RT Master Mix (Takara). qPCR was performed using TB Green Premix Ex Taq (Takara) and a CFX96 Touch Real-Time PCR Detection System (Bio-Rad). GAPDH served as an internal control. The oligonucleotide primers used to carry out the qPCRs were as follows: Yap, sense (5′-GGC​TTG​ACC​CTC​GTT​TTG​C-3′) and antisense (5′-CTG​TGC​TGG​GAT​TGA​TAT​TCC​G-3′); Cyr614, sense (5′-AGG​CTT​CCT​GTC​TTT​GGC​AC-3′) and antisense (5′-ATC​CGG​GTC​TCT​TTC​ACC​AG-3′); Bcl-2, sense (5′-CTG​GGA​TGC​CTT​TGT​GGA​AC-3′) and antisense (5′-AGG​TAT​GCA​CCC​AGA​GTG​ATG-3′); Bcl-xL, sense (5′-ACC​TCC​TCC​CGA​CCT​ATG​ATA​C-3′) and antisense (5′-AGA​AAG​TCA​ACC​ACC​AGC​TCC-3′).

### 2.9 Cell viability MTT assay

rMC-1 cells were incubated with 3-(4,5-Dimethyl-2-thiazolyl)-2,5-diphenyltetrazolium bromide (MTT; Nacalai Tesque) for 3 h at 37°C. After incubation, the medium was removed and DMSO was added to each well. Absorbance was measured at 570 nm using a microplate reader. The viability of cells treated with DMSO was set to 100%.

### 2.10 Statistics

All data are expressed as mean ± SEM. Data were collected from at least three independent experiments. For Western blotting, qPCR, and histological analyses, one eye from each rat was evaluated as a single data point. Statistical analyses were carried out using the Mann-Whitney U test for two groups or one-way Analysis of Variance (ANOVA) followed by Tukey’s or Dunnett’s test for multiple comparisons. Statistical significance was set at *P* < 0.05. All statistical analyses were performed using GraphPad Prism 10 (GraphPad Software Inc., La Jolla, CA, United States).

## 3 Results

### 3.1 YAP is activated in rat Müller cells in response to NMDA-induced RGC injury

**FIGURE 1:**
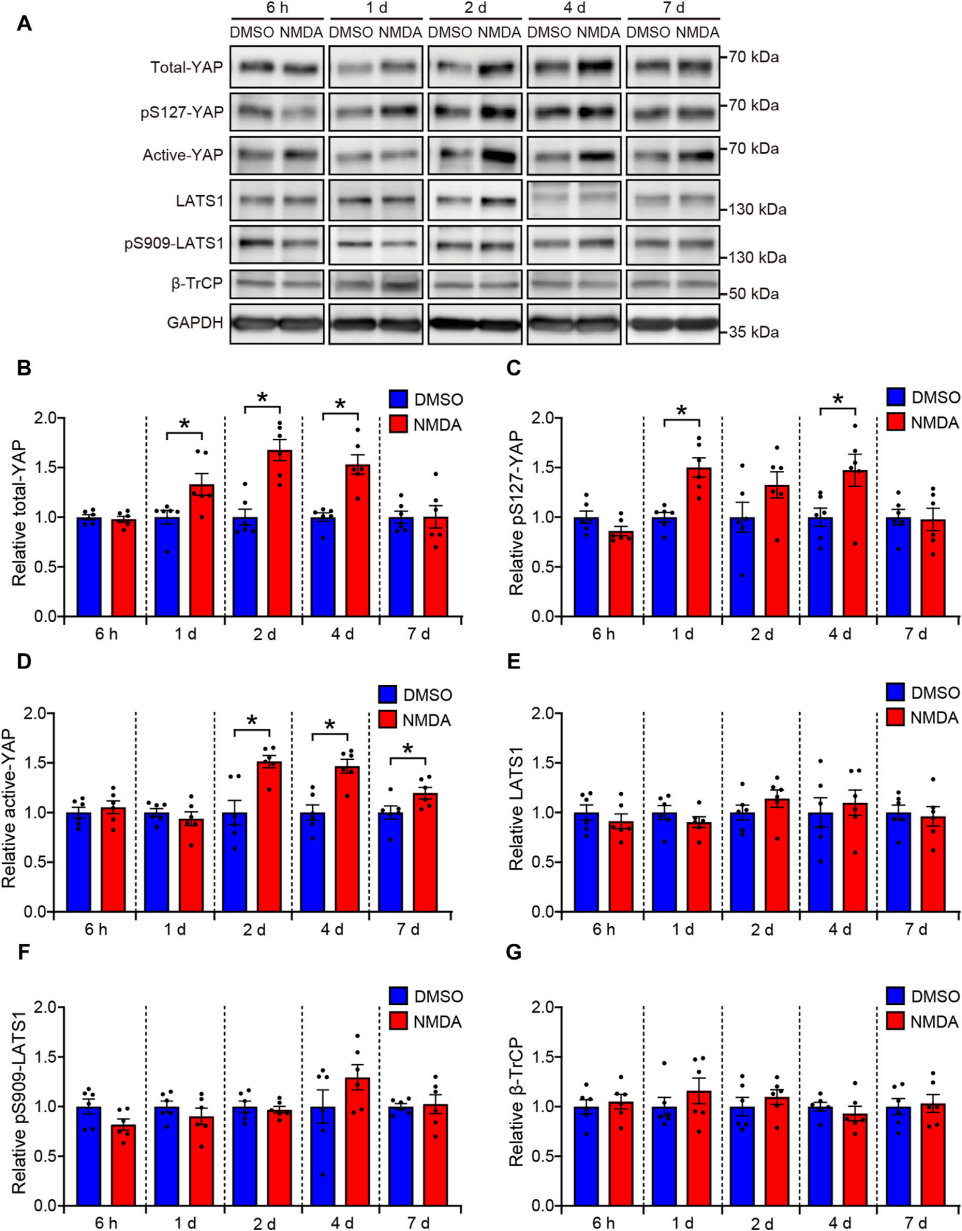
YAP is activated in the NMDA-treated rat retinas. Retinas were harvested at indicated time points after intravitreal injection of DMSO or 200 nmol/eye NMDA. Retina lysates were immunoblotted with indicated antibodies. GAPDH was used as loading control. Representative immunoblots **(A)** and relative protein levels of total-YAP **(B)**, pS127-YAP **(C)**, active-YAP **(D)**, LATS1 **(E)**, pS909-LATS1 **(F)**, and β-TrCP **(G)** at each indicated time point. n = 6 retinas. *P < 0.05 versus each DMSO, by Mann-Whitney U test. Data represent the mean ± SEM.

First, we determined the effect of NMDA-induced RGC injury on the YAP protein levels in rat retinas. The rats were treated intravitreally with DMSO or 200 nmol/eye NMDA and sacrificed at time points ranging from 6 h to 7 days. Total YAP and pS127-YAP protein levels significantly increased 1 day after NMDA injection, peaking at 2–4 days (Figures 1A–C). Active-YAP protein levels significantly increased 2 days after NMDA injection and continued until 7 days (Figures 1A, D), suggesting that YAP was activated in NMDA-treated retinas. Next, we examined the involvement of LATS1 and β-TrCP, which mediate YAP degradation, in the upregulation of YAP expression in NMDA-treated retinas. Protein levels of LATS1, pS909-LATS1, and β-TrCP did not alter after the NMDA injection (Figures 1A, E–G), suggesting that YAP upregulation in NMDA-treated retinas may be independent of Hippo pathway and β-TrCP-mediated YAP degradation.

**FIGURE 2:**
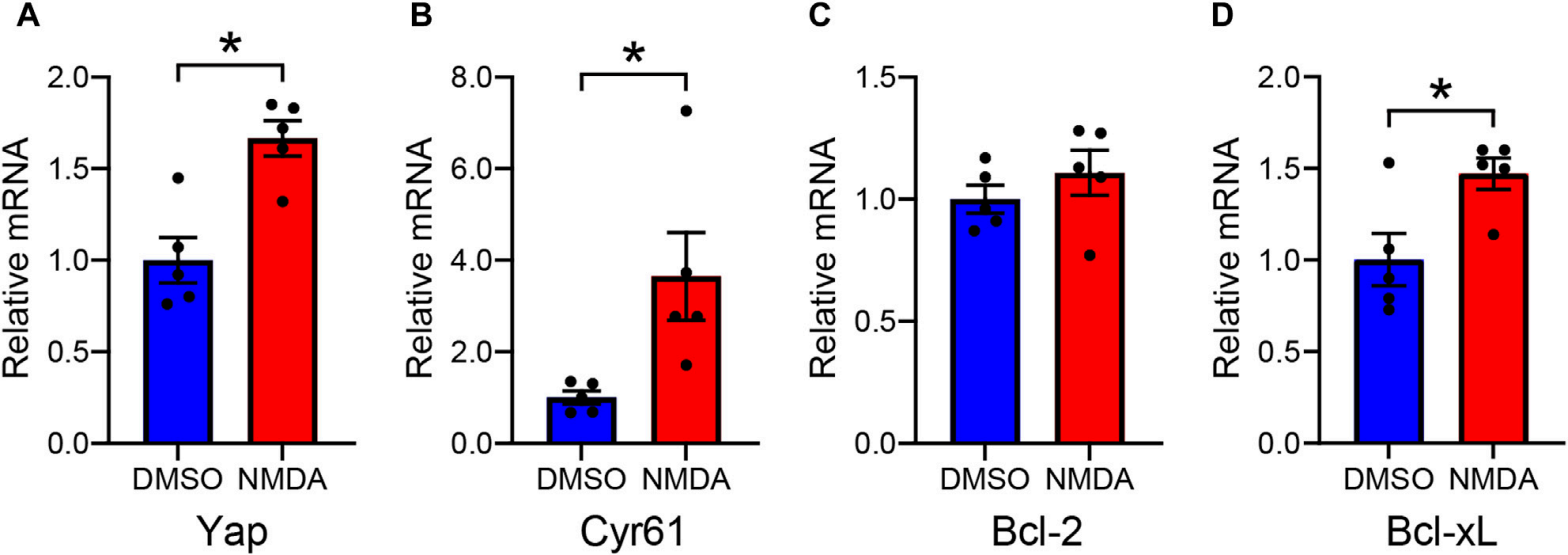
Altered levels of YAP and YAP target gene mRNAs in the DMSO-treated and the NMDA-treated rat retinas. Retinas were collected 2 days after intravitreal injection of DMSO or 200 nmol/eye NMDA. Relative mRNA levels of Yap **(A)**, Cyr61 **(B)**, Bcl-2 **(C)**, and Bcl-xL **(D)** in retinas were evaluated by qPCR. n = 5 retinas. *P < 0.05 versus DMSO, by Mann-Whitney U test. Data represent the mean ± SEM.

We investigated the effect of NMDA-induced RGC injury on *Yap* mRNA levels in rat retinas. In NMDA-treated retinas, *Yap* mRNA levels were significantly higher than in DMSO-treated retinas (Figure 2A). This suggests that the upregulation of YAP observed in NMDA-treated retinas is partly due to an increase in *Yap* mRNA levels. To clarify whether downstream YAP signaling is activated in NMDA-treated retinas, we evaluated the mRNA levels of YAP target genes such as *Cyr61*, *Bcl-2*, and *Bcl-xL*. The mRNA levels of *Cyr61* and *Bcl-xL*, but not *Bcl-2*, in NMDA-treated retinas were significantly higher than those in DMSO-treated retinas (Figures 2B–D). These results suggest the upregulation of YAP signaling after NMDA-induced RGC injury.

**FIGURE 3:**
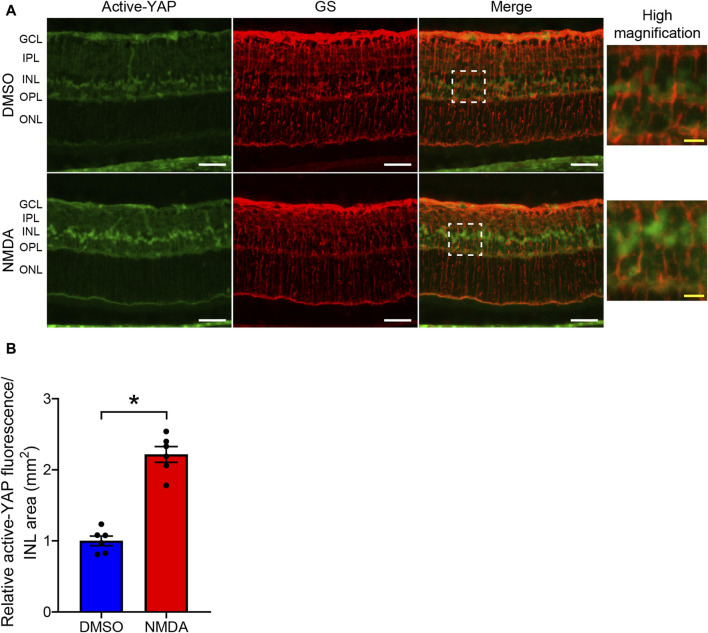
NMDA-induced retinal injury increases YAP activity in Müller cells. **(A,B)** Rat eyes were enucleated 4 days after intravitreal injection of DMSO (upper panels) or 200 nmol/eye NMDA (lower panels). Immunohistochemical analyses were performed using anti-active-YAP antibody and anti-glutamine synthetase (GS) antibody in retinas. Representative images **(A)** and quantification of relative active-YAP fluorescence/INL area (mm^2^) **(B)**. n = 6 eyes. White scale bar: 50 μm; original magnification, ×400. Right side panels show high magnification images of the corresponding white dot boxes in merged images. Yellow scale bar: 10 μm. GCL, ganglion cell layer; IPL, inner plexiform layer; INL, inner nuclear layer; OPL, outer plexiform layer; ONL, outer nuclear layer. *P < 0.05 versus DMSO, by Mann-Whitney U test. Data represent the mean ± SEM.

Previous studies have demonstrated that YAP is specifically expressed in the Müller cells of the murine retina (Hamon et al., 2017; Masson et al., 2020). Therefore, we examined whether YAP was activated in Müller cells after 200 nmol/eye NMDA injection. We compared active-YAP expression levels between NMDA-treated and DMSO-treated retinas 4 days after the injections. Double fluorescent staining of active-YAP and GS, a Müller cell-specific marker, revealed that active-YAP was mainly expressed in the nuclei, microvilli, and endfeet of Müller cells (Figure 3A). In NMDA-treated retinas, active-YAP immunoreactivity in the nuclei of Müller cells was significantly higher than that in DMSO-treated retinas (Figure 3B). These results indicate that YAP is activated in Müller cells after NMDA-induced RGC injury.

### 3.2 Morphological analysis of effects of YAP inhibition by VP in the NMDA-treated rat retina

**FIGURE 4:**
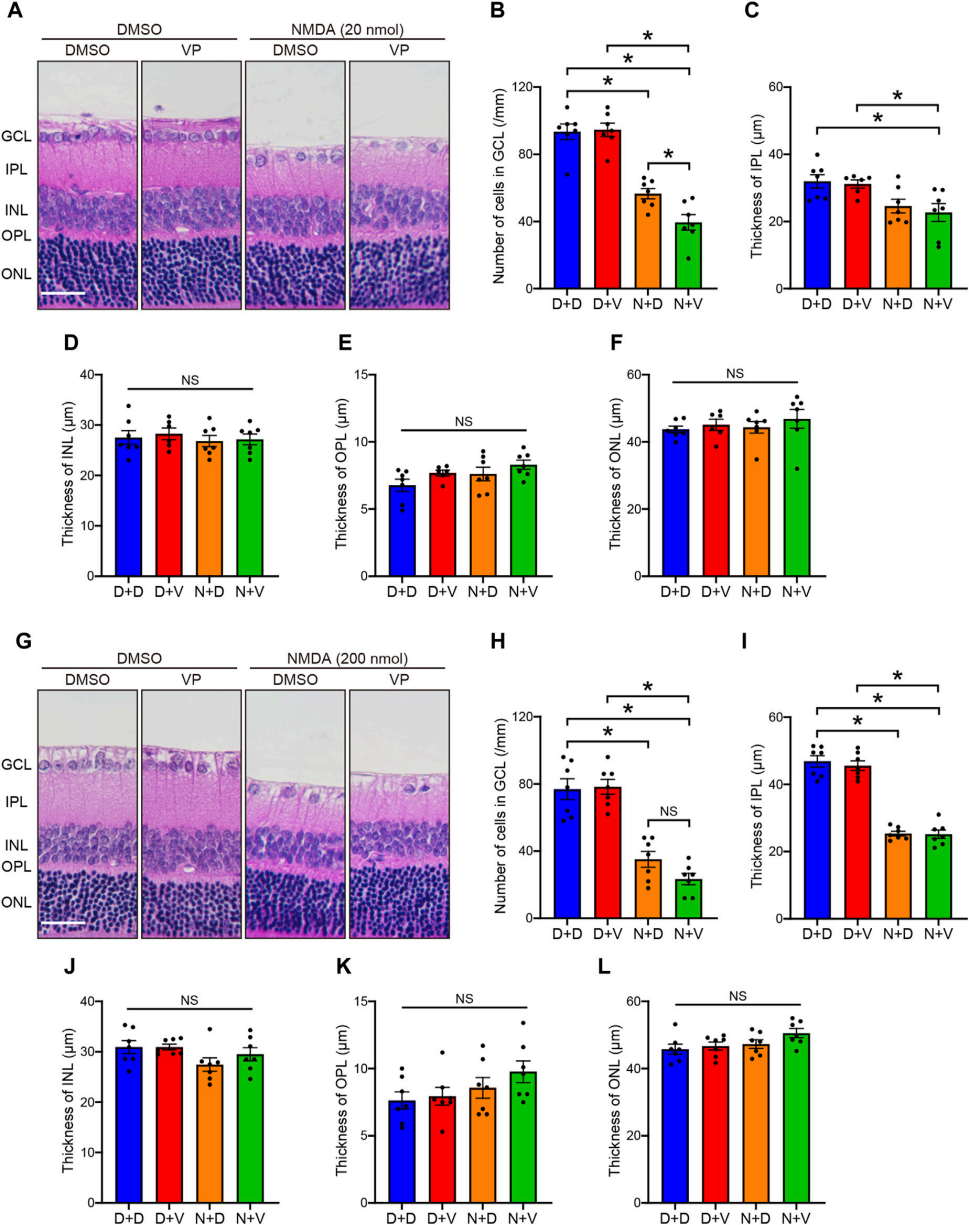
Intravitreal injection of VP decreases RGCs in the NMDA-treated rat retinas. DMSO or NMDA (20 or 200 nmol/eye) was intravitreally injected into both eyes in each rat. Two days after the injection, DMSO or VP (3 nmol/eye) was intravitreally injected into one eye in each rat. Eyes were enucleated 4 days after the first injection. **(A–F)** Effect of VP on 20 nmol/eye NMDA-induced retinal injury. n = 7 eyes. **(G–L)** Effect of VP on 200 nmol/eye NMDA-induced retinal injury. n = 7 eyes. **(A, G)** Representative HE staining images of the eyes treated with DMSO + DMSO (D + D), DMSO + VP (D + V), NMDA + DMSO (N + D), and NMDA + VP (N + V). Scale bar: 50 μm; original magnification, ×200. Number of cells in GCL (cells/mm) **(B, H)**, thickness of IPL (μm) **(C, I)**, thickness of INL (μm) **(D, J)**, thickness of OPL (μm) **(E, K)**, and thickness of ONL (μm) **(F, L)** are shown. GCL, ganglion cell layer; IPL, inner plexiform layer; INL, inner nuclear layer; OPL, outer plexiform layer; ONL, outer nuclear layer; NS, not significant. *P < 0.05, by 1-way ANOVA with Tukey’s test. Data represent the mean ± SEM.

Next, to investigate whether YAP activation in Müller cells exerts salutary or detrimental effects on NMDA-induced RGC injury, we performed a morphological analysis of the effects of YAP inhibition by VP, a selective YAP inhibitor, in NMDA-treated- and DMSO-treated retinas. DMSO or VP (3 nmol/eye) was intravitreally injected into one eye of the rats 2 days after DMSO or NMDA injection, as active-YAP proteins significantly increased 2 days after 20 and 200 nmol/eye NMDA injections (Figures 1A, D; Supplementary Figure S1). The number of cells in the GCL and thickness of the IPL in both the 20 and 200 nmol/eye NMDA + DMSO groups were lower than those in the DMSO + DMSO group (Figures 4A–C, G–I). Importantly, VP slightly reduced the number of cells in the GCL in retinas treated with 200 nmol/eye NMDA, which is the maximum effective dose for RGC loss (Manabe and Lipton, 2003), while VP significantly reduced the number of cells in the GCL in retinas treated with 20 nmol/eye NMDA, which had a weaker effect on RGC loss than 200 nmol/eye NMDA (*P* < 0.05, Mann-Whitney U test) (Figures 4A, B, G, H). The thicknesses of the INL, OPL, and ONL were not significantly different between the four groups (Figures 4D–F, J–L). These results suggest that YAP activation in Müller cells contributes to RGC survival after NMDA-induced RGC injury.

### 3.3 YAP promotes rMC-1 cell survival by upregulating Bcl-xL expression

**FIGURE 5:**
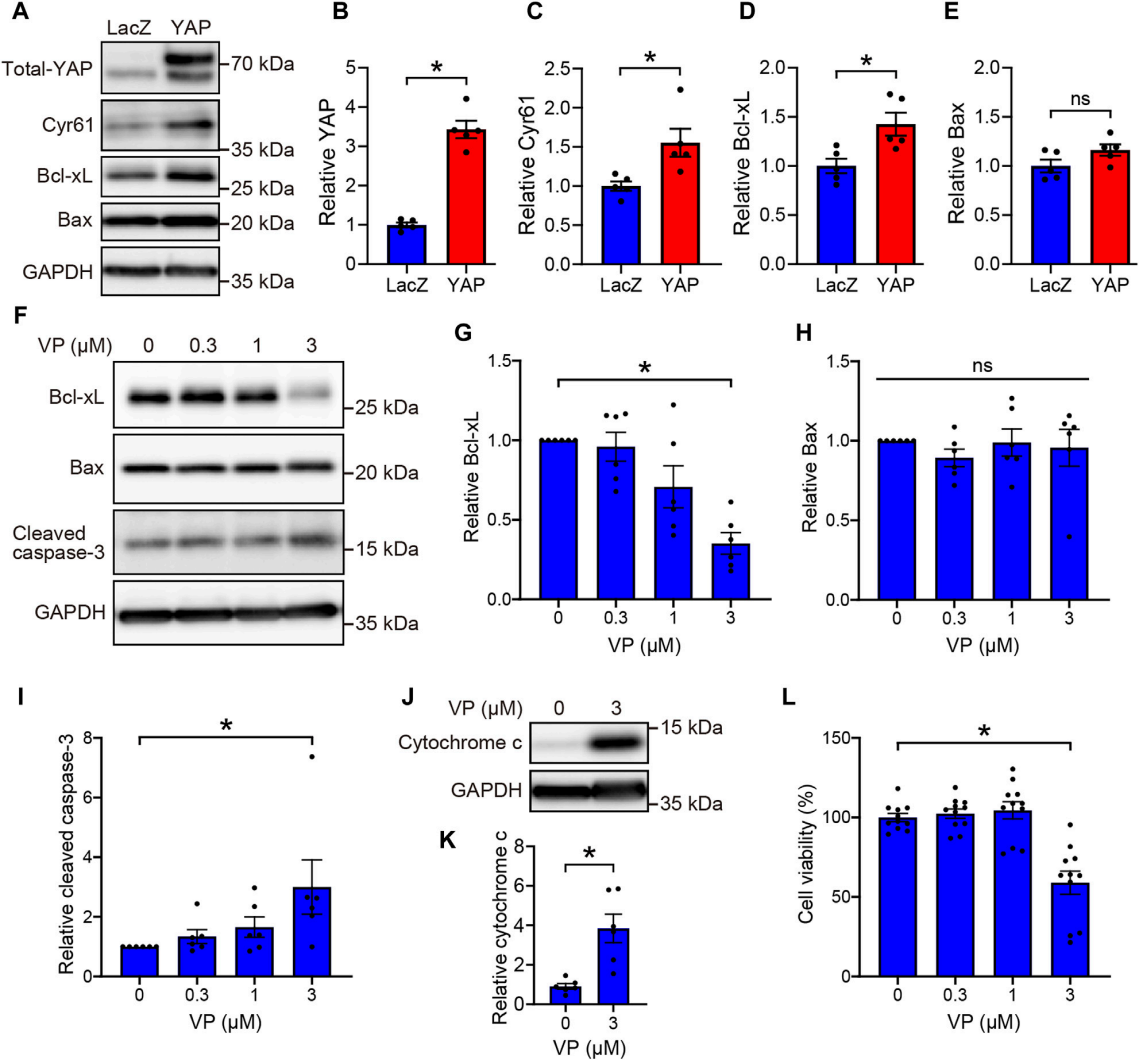
YAP-mediated upregulation of Bcl-xL promotes cell survival in rMC-1 cells. **(A–E)** rMC-1 cells were transduced with Ad-LacZ or Ad-FLAG-YAP for 3 days. Representative immunoblots **(A)** and quantification of results **(B–E)**. n = 5 dishes. *P < 0.05 versus LacZ, by Mann-Whitney U test. (**F**–**L**) rMC-1 cells were treated with DMSO or VP (0.3–3 μM) for 3 days. Representative immunoblots **(F, J)** and quantification of results **(G–I, K)**. Western blotting was performed with indicated antibodies. (**L)** Effect of VP on cell viability of rMC-1 cells. Cell viability was determined using MTT assay. n = 11 wells from three independent experiments. **(K)** *P < 0.05 versus DMSO, by Mann-Whitney U test. **(G–I, L)** *p < 0.05, by 1-way ANOVA with Dunnett’s test. Data represent the mean ± SEM.

YAP is known to promote cell survival by enhancing the expression of Bcl-xL, which inhibits mitochondria-mediated apoptosis (Greenhough et al., 2018; LeBlanc et al., 2018; Tan et al., 2019). To investigate the functional significance of YAP activation in Müller cells, we examined whether YAP regulates cell survival by upregulating Bcl-xL expression in rMC-1 cells. We first determined whether YAP overexpression increased Bcl-xL protein levels in rMC-1 cells transduced with either Ad-LacZ or Ad-YAP. Transduction of rMC-1 cells with Ad-YAP caused approximately 3-fold overexpression of YAP, in accordance with our previous study (Figures 5A, B) (Kashihara et al., 2022). YAP overexpression significantly increased the protein levels of Bcl-xL and Cyr61, but not those of Bax (Figures 5A, C–E). Next, we examined the effect of YAP inhibition on the expression of apoptosis-related factors, such as Bcl-xL, Bax, and cleaved caspase-3 in rMC-1 cells. Interestingly, VP (0.3–3 μM) concentration-dependently decreased protein levels of Bcl-xL and increased cleaved caspase-3 without affecting protein levels of Bax (Figures 5F–I). Since Bcl-xL inhibits cytochrome c release from mitochondria, which is a key initiative step in the apoptosis process (Tsujimoto, 1998), we examined the effect of VP (3 μM) on cytochrome c release in rMC-1 cells. VP (3 μM) markedly increased cytochrome c release from mitochondria (Figures 5J, K) and significantly reduced rMC-1 cell viability (Figure 5L). These results suggest that the YAP-mediated upregulation of Bcl-xL promotes rMC-1cell survival by maintaining mitochondrial integrity.

### 3.4 The expression of Bcl-xL is upregulated in Müller cells from the NMDA-treated rat retina through YAP signaling

**FIGURE 6:**
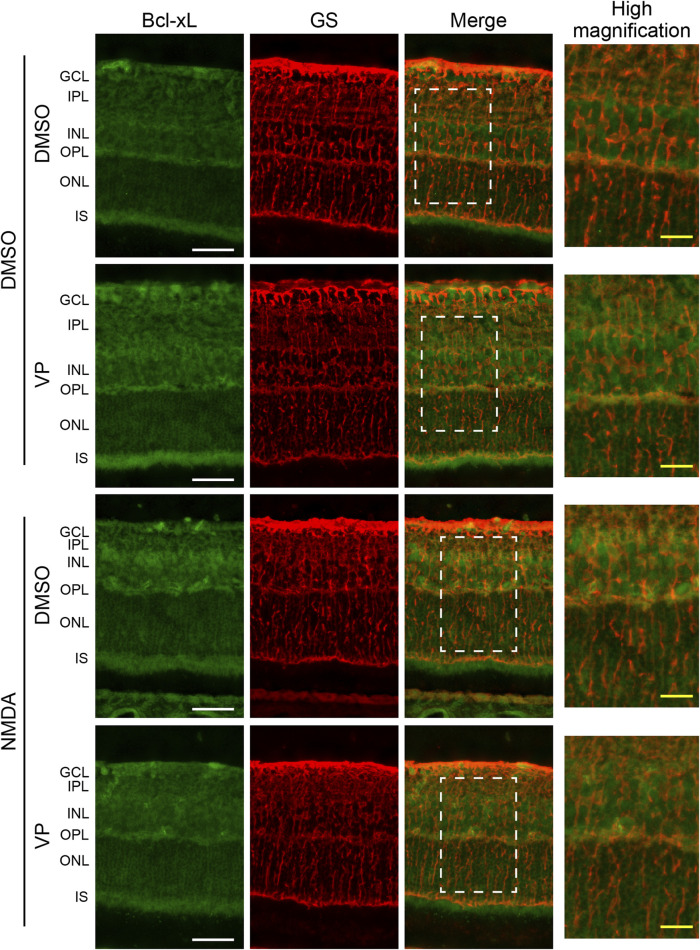
Intravitreal injection of VP suppresses an increase in the expression levels of Bcl-xL in Müller cells from the NMDA-treated rat retinas. DMSO or 200 nmol/eye NMDA was intravitreally injected into both eyes in each rat. Two days after the injection, DMSO or 3 nmol/eye VP was intravitreally injected into one eye in each rat. Eyes were enucleated 4 days after first injection. Immunohistochemical analyses were performed using anti-Bcl-xL antibody and anti-GS antibody in retinas. Representative images of three independent experiments are shown. White scale bar: 50 μm; original magnification, ×400. Right side panels show high magnification images of the corresponding white dot boxes in merged images. Yellow-scale bar: 10 μm. GCL, ganglion cell layer; IPL, inner plexiform layer; INL, inner nuclear layer; OPL, outer plexiform layer; ONL, outer nuclear layer; IS, inner segment of photoreceptors.

To examine whether YAP activation increased the expression levels of Bcl-xL in Müller cells after NMDA-induced RGC injury, we performed double immunofluorescence staining using anti-Bcl-xL and anti-GS antibodies in retinas from the DMSO + DMSO, DMSO + VP (3 nmol/eye), NMDA (200 nmol/eye) + DMSO, and NMDA (200 nmol/eye) + VP (3 nmol/eye) groups. As shown in Figure 6, Bcl-xL immunolabeling was observed in the GCL, IPL, INL, OPL, IS, and ONL. Importantly, the expression levels of Bcl-xL in the NMDA + DMSO group increased primarily in the INL, including the cell bodies of Müller cells, compared to those in the DMSO + DMSO group (Figure 6). Furthermore, a comparison of the results of the NMDA + DMSO and NMDA + VP groups revealed that the intravitreal injection of VP successfully suppressed Bcl-xL upregulation after NMDA-induced RGC injury (Figure 6). These results suggest that NMDA-induced RGC injury stimulates Bcl-xL upregulation in Müller cells via YAP signaling.

### 3.5 Effects of YAP inhibition by VP on Müller cell and RGC apoptosis and oxidative stress in the NMDA-treated rat retina

**FIGURE 7:**
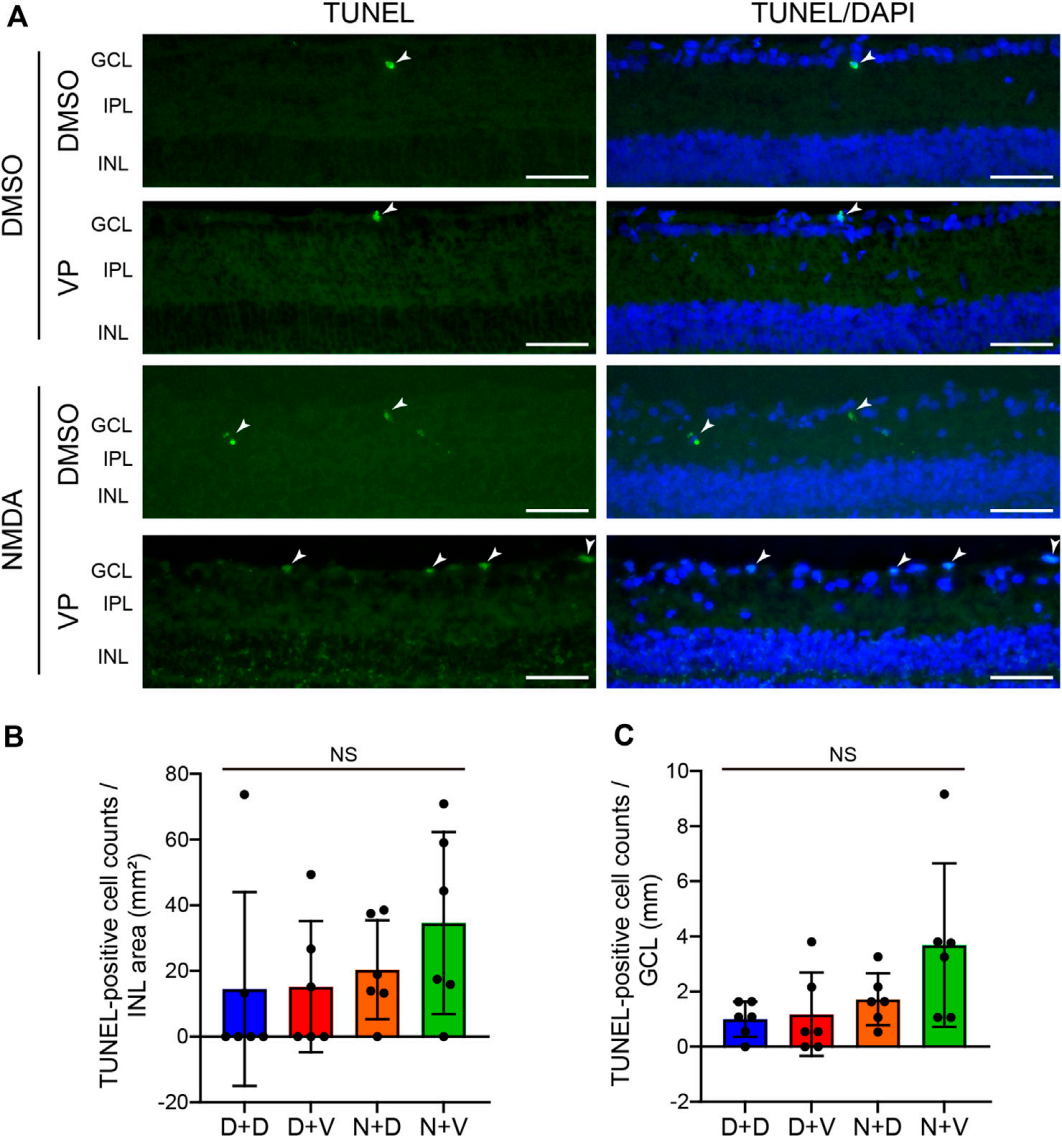
Effects of intravitreal injection of VP on Müller cell and RGC apoptosis in the NMDA-treated rat retinas. **(A–C)** DMSO or 20 nmol/eye NMDA was intravitreally injected into both eyes in each rat. Two days after the injection, DMSO or 3 nmol/eye VP was intravitreally injected into one eye in each rat. Eyes were enucleated 3 days after first injection. TUNEL staining was performed on frozen retinal sections. Representative images of retinal sections labeled with TUNEL (green) and DAPI (blue) **(A)** and quantification of TUNEL-positive cell counts/INL area (mm^2^) **(B)** and TUNEL-positive cell counts/GCL (mm) **(C)**. n = 6 eyes. The arrowhead indicates a TUNEL-positive cell. White scale bar: 50 μm; original magnification, ×400. GCL, ganglion cell layer; IPL, inner plexiform layer; INL, inner nuclear layer. NS, not significant, by one-way ANOVA with Tukey’s test. Data represent the mean ± SEM.

To examine the effect of YAP activation in Müller cells after NMDA-induced RGC injury, we performed TUNEL staining of DMSO-treated and NMDA (20 nmol/eye)-treated retinas with DMSO or VP (3 nmol/eye). DMSO or VP was injected intravitreally into one eye of the rats 2 days after DMSO or NMDA injection, and the eyes were enucleated the following day. In the INL, where Müller cell nuclei are located, almost no TUNEL-positive cells were observed in any of the groups (Figures 7A, B). This suggests that intravitreal injection of DMSO, NMDA (20 nmol/eye), or VP (3 nmol/eye) induces minimal apoptosis in Müller cells. In contrast, in the GCL, where RGC nuclei are located, the NMDA + VP group tended to exhibit increased numbers of TUNEL-positive cells when compared with the NMDA + DMSO group, suggesting that YAP in Müller cells may contribute to the inhibition of RGC apoptosis in NMDA-treated retinas (Figures 7A, C).

**FIGURE 8:**
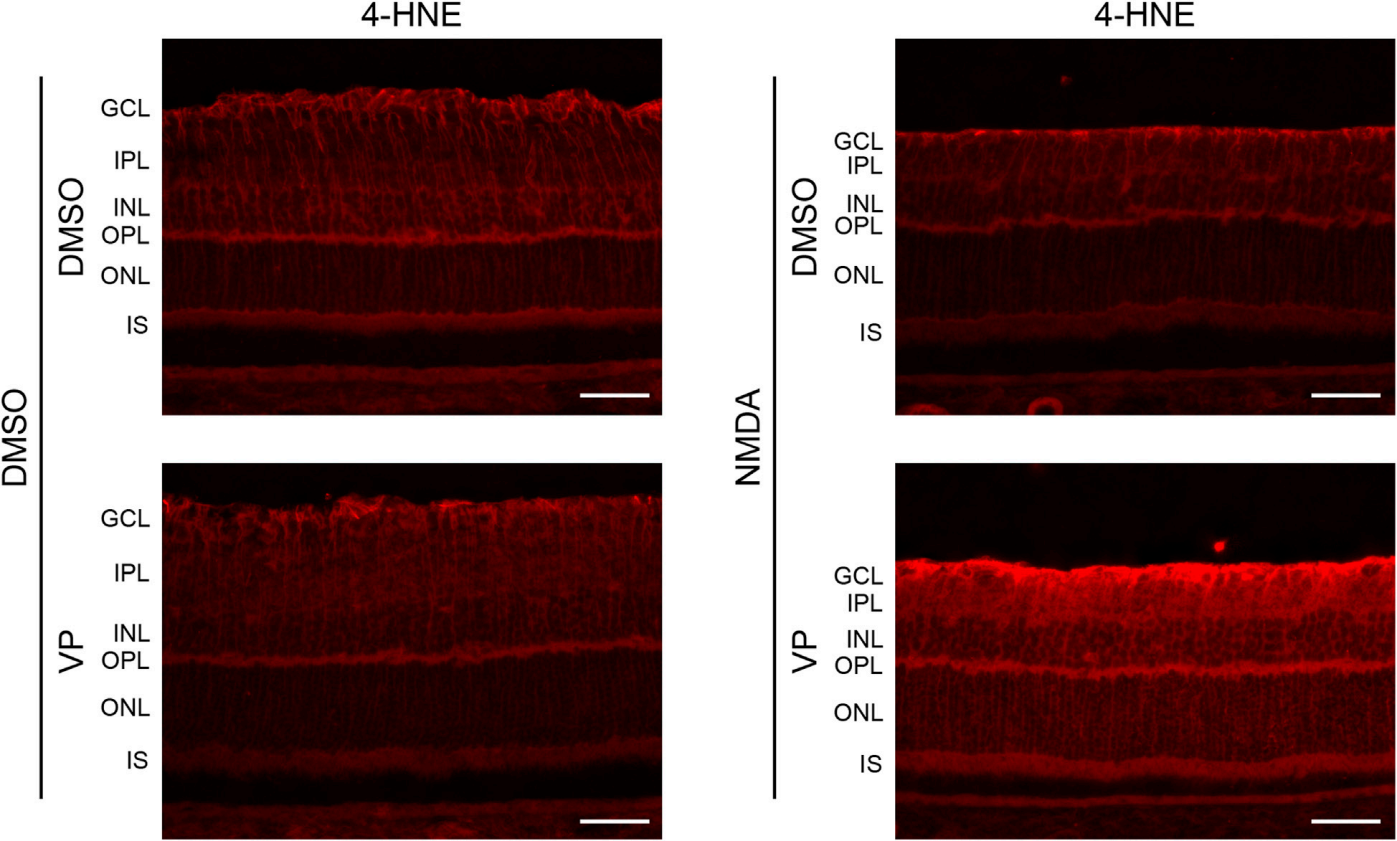
Effects of intravitreal injection of VP on oxidative stress in the NMDA-treated rat retina. DMSO or 20 nmol/eye NMDA was intravitreally injected into both eyes in each rat. Two days after the injection, DMSO or 3 nmol/eye VP was intravitreally injected into one eye in each rat. Eyes were enucleated 3 days after first injection. Immunohistochemical analyses were performed using anti-4-HNE antibody. Representative images of three independent experiments are shown. White scale bar: 50 μm; original magnification, ×400. GCL, ganglion cell layer; IPL, inner plexiform layer; INL, inner nuclear layer; OPL, outer plexiform layer; ONL, outer nuclear layer; IS, inner segment of photoreceptors.

Müller cells regulate the survival of retinal neurons by providing antioxidants (Carpi-Santos et al., 2022). Therefore, we examined whether YAP activation in Müller cells suppressed oxidative stress in the retina after NMDA-induced RGC injury. The expression levels of 4-HNE, a biomarker of oxidative stress, were similar among the DMSO + DMSO, DMSO + VP, and NMDA + DMSO groups (Figure 8). However, the NMDA + VP group exhibited an increase in 4-HNE expression across the entire retina compared with the other groups (Figure 8). The results suggest that YAP activation in Müller cells contributes to the suppression of oxidative stress in the retina and inhibits RGC apoptosis after NMDA-induced RGC injury.

## 4 Discussion

RGC degeneration is a common neurodegenerative disorder of retinal diseases such as glaucoma (Gupta and Yücel, 2007; Schmidt et al., 2008). Previous studies have shown that YAP expressing in Müller cells is involved in retinal neurodegeneration in mice (Hamon et al., 2017; Masson et al., 2020; Yang et al., 2022). However, the function of YAP in Müller cells during RGC degeneration remains unclear. In the present study, we investigated the role of YAP in Müller cells in response to NMDA-induced RGC degeneration by NMDA injection in rats. The major findings of this study are as follows: 1) YAP is increased and activated in Müller cells in response to NMDA-induced RGC injury, 2) pharmacological inhibition of YAP by VP exacerbates NMDA-induced RGC injury, and 3) YAP-mediated upregulation of Bcl-xL promotes Müller cell survival by maintaining mitochondrial integrity. These results suggest that YAP activation in Müller cells plays an important role in preventing RGC degeneration.

Total YAP, activated YAP, and pS127-YAP protein levels increased in the retina after NMDA injection. An increase in activated YAP expression suggests enhanced YAP signaling, whereas an increase in pS127-YAP expression suggests cytosolic retention and degradation of YAP (Yu et al., 2015). To determine whether the increase in YAP expression in response to NMDA-induced RGC injury activated downstream signaling, we measured the mRNA levels of YAP target genes. We found that the mRNA levels of Cyr61, a well-known YAP target gene, and Bcl-xL increased in NMDA-treated retinas. Activated YAP increased markedly in the nuclei of Müller cells in NMDA-treated retinas. Furthermore, YAP overexpression increased the protein levels of Cyr61 and Bcl-xL in rMC-1 cells. The findings suggest that an increase in YAP levels in Müller cells after NMDA-induced RGC injury results in the accumulation of YAP in the nucleus and enhances YAP signaling.

We also investigated the molecular mechanisms underlying the increased YAP expression in Müller cells in response to NMDA-induced RGC injury. YAP is the central downstream effector of the Hippo signaling pathway. Activation of LATS1/2, the core kinases of the Hippo pathway, leads to increased phosphorylation of YAP at Ser127 and inhibition of YAP-mediated gene expression. A recent study suggested that photobiomodulation therapy with 670 nm light decreases phosphorylated LATS1 and activates YAP in cultured mouse Müller cells (Kim et al., 2023). Therefore, the Hippo pathway may be inactivated during NMDA-induced RGC injury. However, no alterations in the expression of LATS1 or phosphorylated LATS1 were observed following the NMDA injection. In cancer cells, decreased expression of β-TrCP, which is involved in the ubiquitin-proteasome system, increases YAP expression (Zhao et al., 2010; Wang et al., 2021). We therefore examined the possibility of downregulation of β-TrCP-dependent YAP degradation in NMDA-treated retinas. Our results show that the expression levels of β-TrCP had no differences between DMSO-treated and NMDA-treated retinas. These results suggest the possibility that the increase in the expression level of activated YAP in Müller cells during RGC degeneration occurs independently of the Hippo pathway and β-TrCP. We further examined whether *Yap* mRNA levels increased in response to NMDA-induced RGC injury. Interestingly, *Yap* mRNA levels increased in the retina 2 days after NMDA injection. Thus, the increased expression of activated YAP may be partially attributed to an increase in *Yap* mRNA. This result is consistent with the findings of the present study, in which not only activated YAP but also total YAP levels increased after NMDA injection.

Intravitreal injection of excessive NMDA selectively damages retinal neurons, such as RGC and amacrine cells that possess NMDA receptors (Lam et al., 1999). Although NMDA-induced RGC loss has been reported to begin approximately 4–6 h after NMDA injection (Lam et al., 1999; Manabe and Lipton, 2003), YAP activation in Müller cells was observed 2 days after NMDA injection. These results raised the question of how NMDA-induced RGC loss leads to YAP activation. We speculate that changes in the retinal structure resulting from RGC loss may stimulate Müller cells, given their role in maintaining the retinal structure (Reichenbach and Bringmann, 2013). Mechanical stress triggers molecular responses in Müller cells (Lindqvist et al., 2010). YAP is also a key mechanotransducer that senses mechanical stress and induces the expression of downstream target genes (Cai et al., 2021). Thus, rather than direct stimulation by NMDA injection, it is reasonable to assume that Müller cells are secondarily stimulated by RGC injury, which leads to YAP activation. Further investigation is required to elucidate the details of this mechanism.

We investigated whether activated YAP in Müller cells protected or exacerbated retinal neurons in response to NMDA (20 or 200 nmol/eye)-induced RGC injury by examining the effects of VP, a selective YAP inhibitor that inhibits YAP-TEAD interactions (Liu-Chittenden et al., 2012; Giraud et al., 2020). VP significantly reduced the number of surviving RGCs treated with 20 nmol/eye NMDA injection, while VP slightly reduced the number of surviving RGCs with 200 nmol/eye NMDA. Intravitreal injection of 200 nmol/eye NMDA represents the maximal dose for inducing RGC loss in adult rats (Manabe and Lipton, 2003). Therefore, VP may fail to reduce the surviving RGCs at 200 nmol/eye NMDA and exacerbate 20 nmol/eye NMDA-induced RGC loss, which exhibited weaker damaging effects on RGCs than 200 nmol/eye NMDA. The results suggest that YAP activation in Müller cells exerts a protective effect on RGC damage induced by NMDA. The results are consistent with previous reports demonstrating the involvement of YAP in Müller cells in retinal neuroprotection (Masson et al., 2020; Lourenço et al., 2021; Yang et al., 2022; Kim et al., 2023).

How does YAP activation in Müller cells suppress the NMDA-induced RGC damage? In the retina, Müller cells play a crucial role in maintaining retinal homeostasis by secreting neurotrophic factors and antioxidants, and regulating glutamate metabolism and ion homeostasis (García and Vecino, 2003; Bringmann et al., 2006; Reichenbach and Bringmann, 2013; Boia et al., 2020). Thus, dysfunction of Müller cells has been implicated as one of the causes of retinal diseases (Bringmann and Wiedemann, 2012; Carpi-Santos et al., 2022). YAP increases the expression of the pro-survival factor Bcl-xL, which inhibits the activation of the pro-apoptotic factor Bax and preserves mitochondrial outer membrane integrity (Greenhough et al., 2018; LeBlanc et al., 2018; Tan et al., 2019). Therefore, we investigated whether the protective effect of YAP on RGCs was mediated by the suppression of mitochondrial impairment via Bcl-xL induction. An increase in the expression of Bcl-xL was primarily observed in the INL of the retina, which contains the cell bodies of Müller cells, after NMDA injection. Furthermore, we examined whether YAP contributed to Müller cell survival by inducing Bcl-xL expression, thereby suppressing mitochondrial impairment in rMC-1 cells. While YAP overexpression increased the expression of Bcl-xL, inhibition of YAP by VP decreased the expression of Bcl-xL in a concentration-dependent manner, increased the release of cytochrome c, and decreased rMC-1 survival rate. In addition, intravitreal injection of VP induced minimal Müller cell apoptosis, but tended to increase oxidative stress in the retina and RGC apoptosis after NMDA-induced RGC injury. These findings suggest that YAP activation in Müller cells in response to NMDA-induced RGC injury may contribute to the maintenance of Müller cell function and protect retinal nerves by suppressing mitochondrial impairment.

In summary, we demonstrated that YAP in Müller cells is activated in response to NMDA-induced RGC injury, and that activated YAP exerts a protective effect against RGC injury by upregulating Bcl-xL expression in Müller cells. This study suggests a salutary role for YAP in Müller cells in RGC degeneration and suggests that YAP is a potential therapeutic target for retinal neurodegenerative diseases.

## Data Availability

The original contributions presented in the study are included in the article/Supplementary Material, further inquiries can be directed to the corresponding authors.

## Abbreviations

RGC, Retinal ganglion cell; YAP, Yes-associated protein 1; NMDA, *N*-methyl-D-aspartic acid; rMC-1, rat Müller cell line; DMSO, Dimethyl sulfoxide; VP, Verteporfin; PFA, Paraformaldehyde; PBS, Phosphate-buffered saline; OCT, Optimum cutting temperature; active-YAP, active form of YAP; GS, Glutamine synthetase; CST, Cell signaling technology; 4-HNE, 4-hydroxy-2-nonenal; TUNEL, Terminal deoxynucleotidyl transferase-mediated deoxyuridine triphosphate nick-end labeling; HE, Hematoxylin and eosin; GCL, Ganglion cell layer; IPL, Inner plexiform layer; INL, Inner nuclear layer; OPL, Outer plexiform layer; IS, Inner segment of photoreceptors; Ad-YAP, FLAG-YAP adenovirus; Ad-LacZ, LacZ control adenovirus; FBS, Fetal bovine serum; β-TrCP, β-transducin repeat-containing protein; GAPDH, Glyceraldehyde3-phosphate dehydrogenase; HRP, Horseradish peroxidase; MTT, 3-(4,5-Dimethyl-2-thiazolyl)-2,5-diphenyltetrazolium bromide.
