# Supplementary material for: YAP activation in Müller cells protects against NMDA-induced retinal ganglion cell injury by regulating Bcl-xL expression
Source: Front Pharmacol. 2024 Aug 6;15:1446521. doi: 10.3389/fphar.2024.1446521 (PMC11333228; doi:10.3389/fphar.2024.1446521)

Full unedited image for Figure 1A_total-YAP_6h

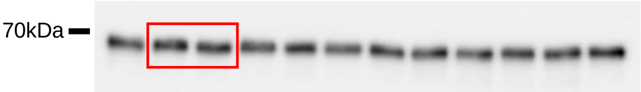

Full unedited image for Figure 1A_total-YAP_1d

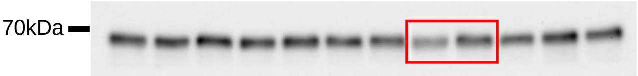

Full unedited image for Figure 1A_total-YAP_2d

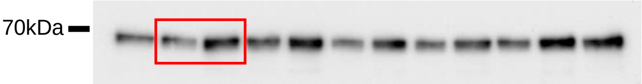

Full unedited image for Figure 1A_total-YAP_4d

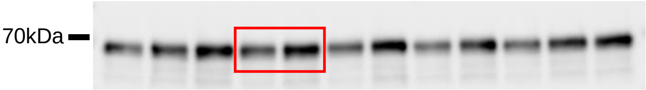

Full unedited image for Figure 1A_total-YAP_7d

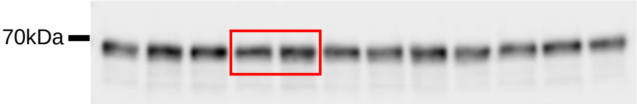

Full unedited image for Figure 1A_pS127-YAP_6h

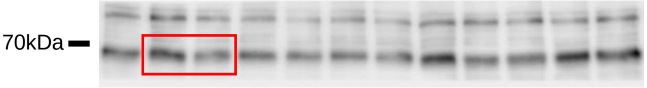

Full unedited image for Figure 1A_pS127-YAP_1d

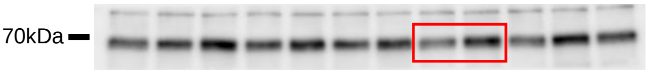

Full unedited image for Figure 1A_pS127-YAP_2d

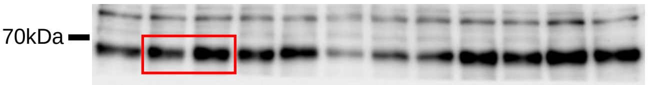

Full unedited image for Figure 1A_pS127-YAP_4d

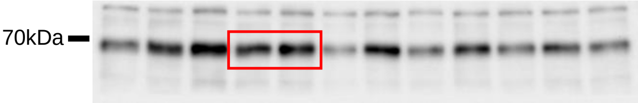

Full unedited image for Figure 1A_pS127-YAP_7d

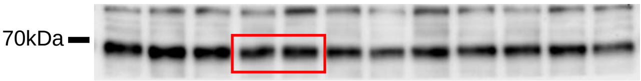

Full unedited image for Figure 1A_active-YAP_6h

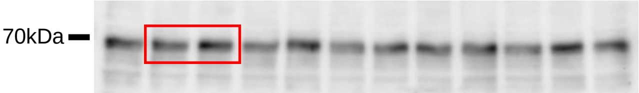

Full unedited image for Figure 1A_active-YAP_1d

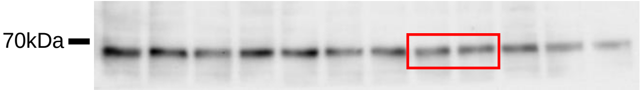

Full unedited image for Figure 1A_active-YAP_2d

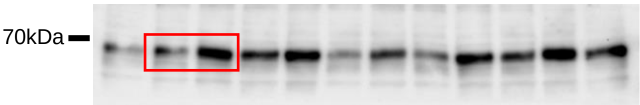

Full unedited image for Figure 1A_active-YAP_4d

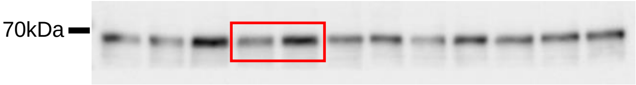

Full unedited image for Figure 1A_active-YAP_7d

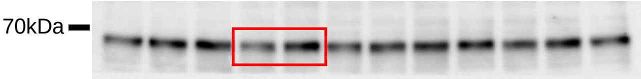

Full unedited image for Figure 1A_LATS1_6h

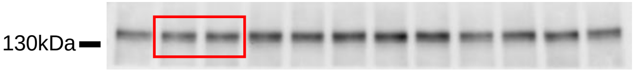

Full unedited image for Figure 1A_LATS1_1d

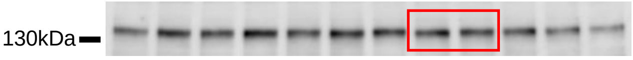

Full unedited image for Figure 1A_LATS1_2d

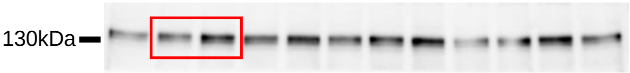

Full unedited image for Figure 1A_LATS1_4d

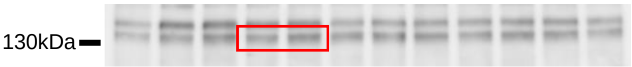

Full unedited image for Figure 1A_LATS1_7d

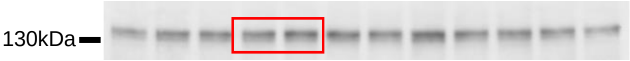

Full unedited image for Figure 1A_pS909-LATS1_6h

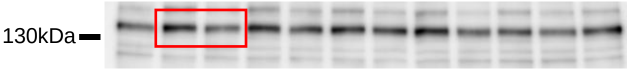

Full unedited image for Figure 1A_pS909-LATS1_1d

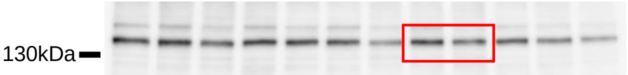

Full unedited image for Figure 1A_pS909-LATS1_2d

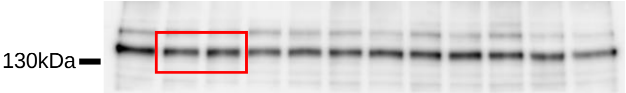

Full unedited image for Figure 1A_pS909-LATS1_4d

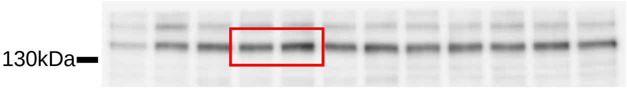

Full unedited image for Figure 1A_pS909-LATS1_7d

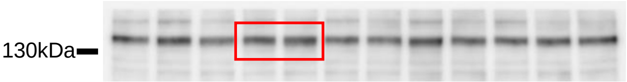

Full unedited image for Figure 1A_β-TrCP_6h

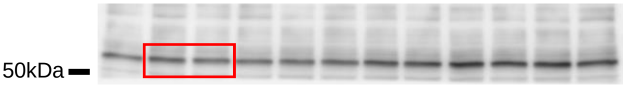

Full unedited image for Figure 1A_ β-TrCP_1d

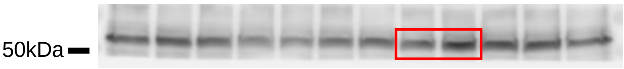

Full unedited image for Figure 1A_ β-TrCP_2d

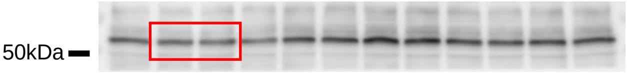

Full unedited image for Figure 1A_ β-TrCP_4d

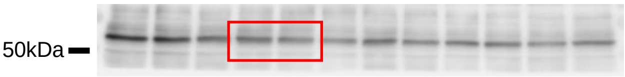

Full unedited image for Figure 1A_ β-TrCP_7d

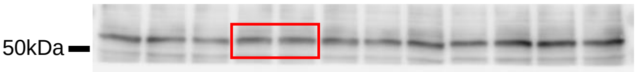

Full unedited image for Figure 1A_GAPDH_6h

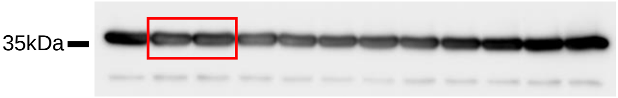

Full unedited image for Figure 1A_ GAPDH_1d

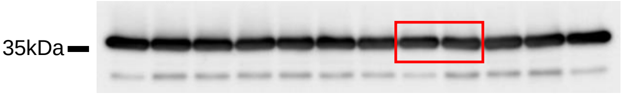

Full unedited image for Figure 1A_ GAPDH_2d

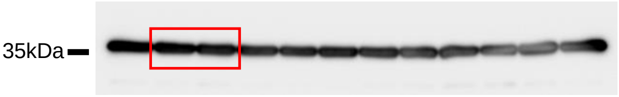

Full unedited image for Figure 1A_ GAPDH_4d

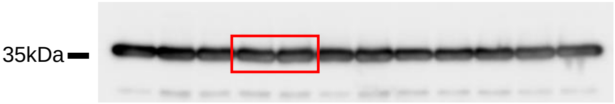

Full unedited image for Figure 1A_ GAPDH_7d

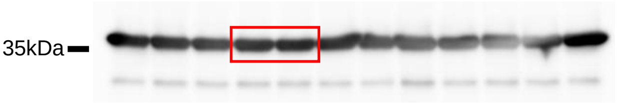

Full unedited image for Figure 5A_total-YAP

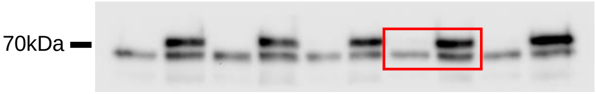

Full unedited image for Figure 5A_Cyr61

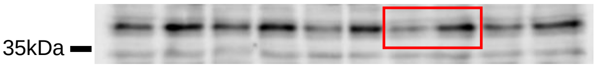

Full unedited image for Figure 5A_Bcl-xL

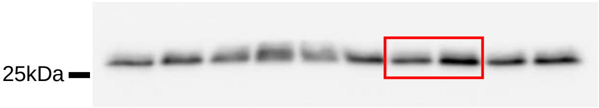

Full unedited image for Figure 5A_Bax

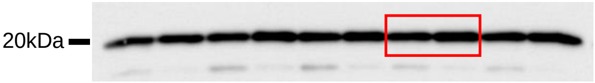

Full unedited image for Figure 5A_GAPDH

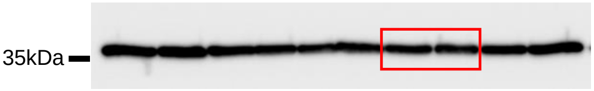

Full unedited image for Figure 5E_Bcl-xL

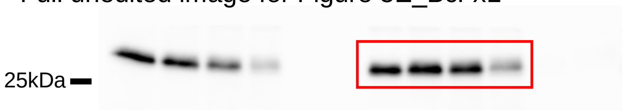

Full unedited image for Figure 5E_Bax

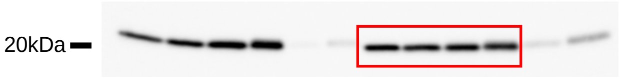

Full unedited image for Figure 5E_Cleaved caspase-3

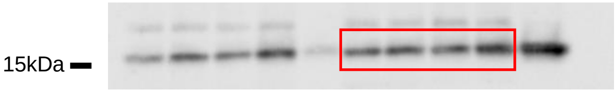

Full unedited image for Figure 5E_GAPDH

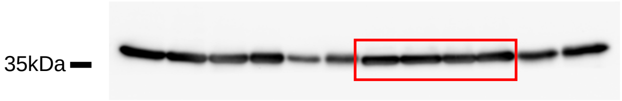

Full unedited image for Figure 5I_cytochrome c

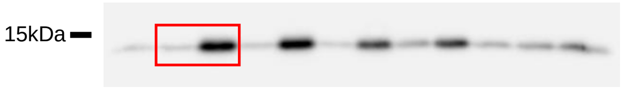

Full unedited image for Figure 5I_GAPDH

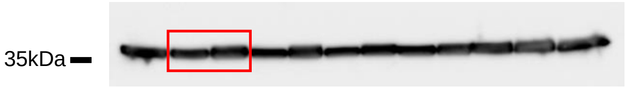

Supplement: Supplementary file 1 [file DataSheet2.PDF]
