# Supplementary material for: YAP activation in Müller cells protects against NMDA-induced retinal ganglion cell injury by regulating Bcl-xL expression
Source: Front Pharmacol. 2024 Aug 6;15:1446521. doi: 10.3389/fphar.2024.1446521 (PMC11333228; doi:10.3389/fphar.2024.1446521)
Supplement: Supplementary file 2 [file DataSheet1.PDF]

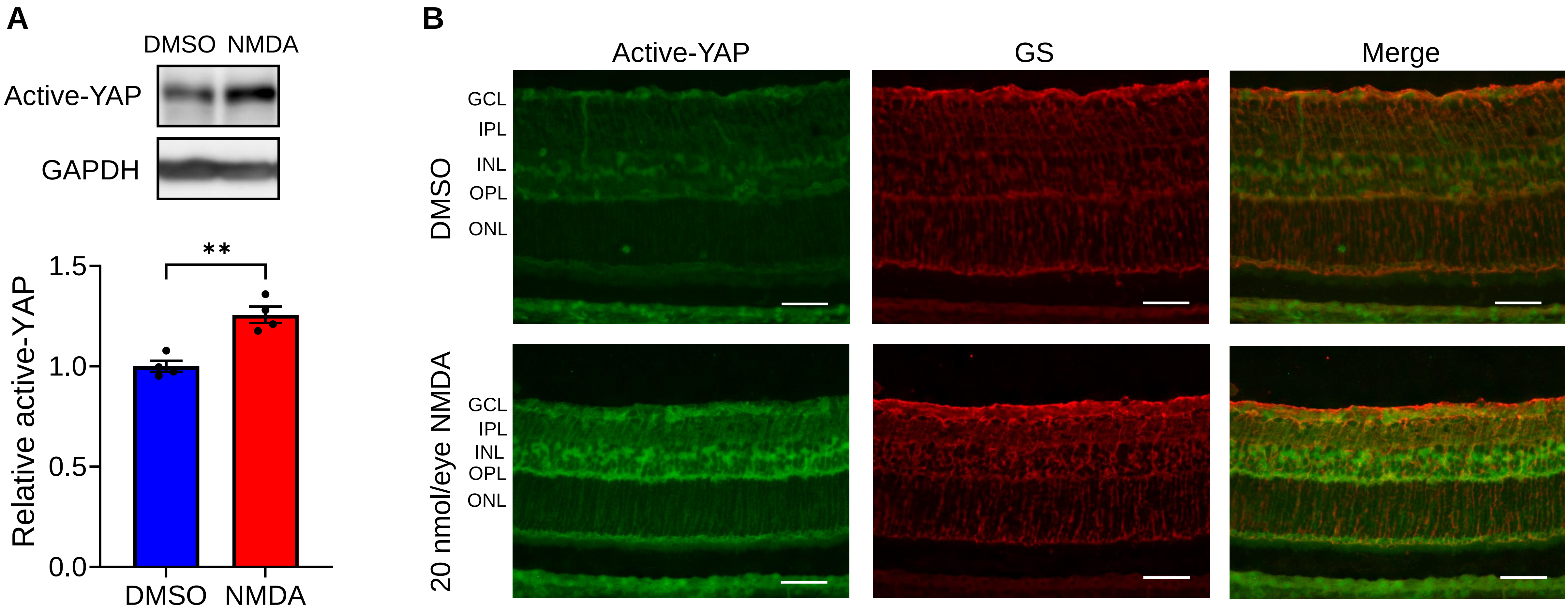

Supplementary Figure S1. 20 nmol/eye NMDA-induced retinal injury activates YAP in Müller cells.
Rat eyes were enucleated 2 days after intravitreal injection of DMSO or 20 nmol/eye NMDA. (**A**) Retina lysates were immunoblotted with active-YAP and GAPDH antibodies. GAPDH was used as loading control. Representative immunoblots and relative protein levels of active-YAP. n = 4 retinas. *P<0.05 versus DMSO, by Mann-Whitney U test. Data represent the mean ± SEM. (**B**) Immunohistochemical analyses were performed using anti-active-YAP antibody and anti-glutamine synthetase (GS) antibody in retinas. Representative images of three independent experiments are shown. White scale bar: 50 μm; original magnification, x400. GCL, ganglion cell layer; IPL, inner plexiform layer; INL, inner nuclear layer; OPL, outer plexiform layer; ONL, outer nuclear layer.
